# Solar Light-Driven Efficient Degradation of Organic Pollutants Mediated by S-Scheme MoS_2_@TiO_2_-Layered Structures

**DOI:** 10.3390/nano15010028

**Published:** 2024-12-27

**Authors:** Wajeehah Shahid, Faryal Idrees, Ji-Jun Zou, Jeong Ryeol Choi, Lun Pan

**Affiliations:** 1 Department of Physics, The University of Lahore, Lahore 54000, Pakistan; wajeehah.shahid@phys.uol.edu.pk; 2 Department of Physics, University of the Punjab, Lahore 54590, Pakistan; 3 Key Laboratory for Green Chemical Technology of the Ministry of Education, School of Chemical Engineering and Technology, Tianjin University, Tianjin 300072, China; jj_zou@tju.edu.cn (J.-J.Z.); panlun76@tju.edu.cn (L.P.); 4 School of Electronic Engineering, Kyonggi University, Suwon 16227, Republic of Korea; 5 Haihe Laboratory of Sustainable Chemical Transformations, Tianjin 300192, China

**Keywords:** photocatalysis, degradation, heterostructures, MoS_2_, TiO_2_, solar light

## Abstract

This study focuses on achieving high photocatalytic activity using MoS_2_/TiO_2_ heterostructures (MOT). To this end, MoS_2_ and TiO_2_ were synthesized by employing hydrothermal synthesis techniques, and then MoS_2_/TiO_2_ heterostructures were synthesized by using 1:1, 1:2, 1:3, and 1:4 ratios of MoS_2_ and TiO_2_, respectively. While the structural and electronic changes for the 1:2 and 1:3 ratios were relatively minor, significant modifications in bandgaps and morphology were observed for the 1:1 and 1:4 ratios. Thus, this study presents a comparative analysis of the photocatalytic performance of the 1:1 (MOT11) and 1:4 (MOT14) heterostructures. The formation of these heterostructures was confirmed through Energy-Dispersive X-ray Spectroscopy (EDX) and Fourier Transform Infrared Spectroscopy (FTIR) analysis. Notably, the bandgaps of MOT11 and MOT14 were red-shifted to 1.66–1.25 eV and 1.01–1.68 eV, respectively, indicating improved visible-light absorption capabilities. The photocatalytic activity of MOT11 and MOT14 was evaluated through the degradation of Rhodamine B (RhB) under simulated solar irradiation. MOT11 demonstrated a high degradation efficiency of 98.9% within 60 min, while MOT14 achieved 98.21% degradation after 90 min of irradiation. The significance of this study lies in its demonstration that a facile synthesis route and a small proportion of MoS_2_ in the heterostructure can achieve excellent photocatalytic degradation performance under solar light. After MS-analysis, S-Scheme has been suggested, which has also been complimented by the scavenger tests. Additionally, the improved photocatalytic properties of MOT11 and MOT14 suggest their potential for future applications in hydrogen generation and water splitting, offering a pathway towards sustainable and clean energy production.

## 1. Introduction

Coping with the rapidly growing global energy demand while transitioning from depleting fossil fuels to sustainable and clean energy sources is a critical challenge. A promising solution in this direction is photocatalytic degradation, which uses sunlight to drive chemical reactions that break down pollutants in water and air, offering an effective way to combat environmental pollution. This process has attracted significant attention due to its potential to degrade a wide range of organic contaminants, including dyes and pharmaceuticals, on a large scale. Photocatalytic degradation presents an eco-friendly alternative to conventional wastewater treatment methods, providing a sustainable approach to environmental remediation and contributing to cleaner ecosystems [1,2,3].

Recently, for efficient photocatalysts, a wide range of ultraviolet (UV) light active materials, e.g., TiO_2_ [1,2], ZnO [3], Nb_2_O_5_ [4], and WO_3_ [5], and visible-light active materials, e.g., g-C_3_N_4_ [6], Co_3_O_4_ [7], MoS_2_ [8], CdS [9], have been investigated. However, UV constitutes only 4–5% of the solar spectrum [10,11], greatly limiting the photocatalysts’ ability to harness sunlight efficiently. Some visible active materials face challenges like poor stability and toxicity, making them less suitable for practical applications. This has highlighted the need for photocatalysts that should not only be active under visible light but should also demonstrate efficient electron–hole separation, high chemical and physical stability, low cost, and non-toxicity [12,13].

Finding a single material with all these properties is challenging, prompting researchers to explore hybrid systems that combine the complementary features of two materials. One such combination is titanium dioxide (TiO_2_) and molybdenum disulfide (MoS_2_). TiO_2_ is a widely used, cost-effective n-type semiconductor known for its stability, but in most cases, synthesized TiO_2_ has a large bandgap that limits its photocatalytic activity to the UV region, which diminishes its photocatalytic response to the full solar spectrum [10]. Conversely, MoS_2_ is a two-dimensional transition metal dichalcogenide (TMDC) with excellent chemical stability and light-absorption capabilities that extend into the visible and infrared regions [13,14,15,16]. MoS_2_ also has a tunable bandgap (ranging from 1.3 to 1.9 eV as it reaches monolayer thickness), making it an effective co-catalyst for enhancing the photocatalytic activity of TiO_2_ [16,17,18,19].

The MoS_2_/TiO_2_ heterojunction system presents an opportunity to leverage the advantages of both materials. This combination can achieve efficient charge carrier separation and migration, minimize electron–hole recombination, and expand light absorption across a broader spectrum. These characteristics suggest that MoS_2_/TiO_2_ could exhibit enhanced photocatalytic performance, making it suitable for applications like hydrogen production and pollutant degradation [20,21,22].

This study aims to achieve high photocatalytic efficiency by engineering MoS_2_-TiO_2_ (MOT) heterostructures. The synthesis of these heterostructures was conducted using a novel hydrothermal method, producing agglomerated hexagonal MoS_2_ and layered TiO_2_ nanostructures. The materials were combined in different ratios (1:1, 1:2, 1:3, and 1:4), with significant changes in morphology and photocatalytic performance observed for the 1:1 and 1:4 mixtures. The successful formation of the heterostructures was confirmed through XRD, EDX, and FTIR analyses, and their photocatalytic performance was evaluated through the degradation of Rhodamine B (RhB). The results demonstrated a notably high reaction rate constant for the photocatalytic degradation of RhB, indicating superior efficiency. Additionally, an S-scheme photocatalytic mechanism was proposed to explain the improved performance, supported by Mott–Schottky analysis to understand the band alignment between MoS_2_ and TiO_2_. The scavenger test helps to further our understanding of the proposed photocatalytic mechanism. This investigation highlights the potential of MoS_2_/TiO_2_ heterostructures to offer a practical solution for clean energy generation and environmental remediation.

## 2. Experimental Section

### 2.1. Materials and Methods

Ammonium Thiocyanate (NH_4_SCN, ≥97.5%) deionized water was obtained by the lab water plant (density, 1.0 g/cm^3^), hydrochloric acid (HCl, 37%), Molybdenium Oxide (MoO_3_, ≥99.5%). All the chemicals used in this study were of analytical grade and were purchased from Sigma Aldrich (St. Louis, MO, USA) and used without further purification.

### 2.2. Synthesis of MoS_2_

MoS_2_ was synthesized using a one-step hydrothermal method, starting with a MoO_3_ and ammonium thiocyanate (NH_4_SCN). Specifically, 1.5 mmol MoO_3_ (0.22 g) and 4.5 mmol NH_4_SCN (0.45 g) were dissolved in 40 mL of deionized water and sonicated for 30 min. The pH of the solution was adjusted to 1 by adding 1 M HCl solution and stirring for 30 min. The obtained homogenous solution was transferred to a 50 mL Teflon-lined stainless-steel autoclave for hydrothermal treatment at 180 °C for 12 h. After cooling to room temperature, the black powder of MoS_2_ was obtained by centrifugation for 10–15 min, followed by multiple washes with deionized water and ethanol. The samples were then dried in an oven at 80 °C for 12 h.

### 2.3. Synthesis of TiO_2_

TiO_2_ nanostructures were synthesized using a hydrothermal technique which has also been reported by our group. The synthesis process is restated as follows: 4 mL of titanium tetraisopropoxide (TTIP, 97%) and 20 mL of absolute ethanol (C_2_H_6_O, 99.8%) were mixed and stirred at 250 °C for 1 h. A mixture of 4 mL of HCl with 1 M concentration was added dropwise to the obtained homogenous solution. The resulting solution was further stirred on a hot plate for 1 h before being transferred to a Teflon-lined stainless-steel autoclave and kept in an oven at 180 °C for 12 h. After hydrothermal treatment, the sealed autoclave was cooled to room temperature, and a white powder was obtained by centrifugation for 10–15 min. The resulting powder was then washed several times with deionized water and ethanol and then dried in an oven at 80 °C for 12 h. Finally, the resulting powder was annealed at 550 °C in a muffle furnace to extract the TiO_2_ nanostructures. A yellowish-white powder was obtained as a result.

### 2.4. Synthesis of MoS_2_-TiO_2_ Heterostructures

Already prepared MoS_2_ and TiO_2_ were used for MOT11 (1:1) and MOT14 (1:4) synthesis by using an ex situ technique. In a typical process, the required amount of MoS_2_ was added to 20 mL of ethanol and continuously stirred to prepare a homogenous solution. Similarly, TiO_2_ was added to 20 mL of ethanol and continuously stirred to prepare a homogenous solution. The prepared MoS_2_ was added dropwise to the TiO_2_ solution while undergoing continuous stirring and the obtained solution was stirred for an additional 1h. After stirring, a viscous solution was obtained which was centrifuged and washed several times with deionized water. The obtained product was dried in an oven at 80 °C for 24 h. Grayish and light-grayish powders were obtained for MOT11 and MOT14, respectively.

### 2.5. Characterization Techniques

XRD samples were measured by JDX-3532, JEOL, Japan, 20–40 kV, 2.5–30 mA, CuKa (Wavelength = 1.5418 Å, 2Theta-Range: 0 to 160°). JSM5910, FESEM samples were measured by JEOL, Tokyo, Japan, 30 kV, 300,000×, Max Resolving power 2.3 nm, Attachments: SEI Detector, Energy-Dispersive X-Ray Spectrometer (EDX), INCA200/Oxford instruments, Oxford, UK (the analysis range is Boron to Uranium). TEM samples were measured by JEM-2100, JEOL, Japan, 200 kV, Magnification (Max): 1,500,000×, Resolving power (Max): 1.4 Å. UV were measured through Shimadzu UV-1800, Kyoto, Japan. The “Abet Technologies Sunlight TM Solar Simulator” (Milford, CT, USA) was used to evaluate the photocatalytic activity. An electrochemical workstation (CS350M, CORRTEST, Wuhan, China) accompanied a reactor and a 450 W xenon lamp was used as a light source.

### 2.6. Photocatalytic Activity

The photocatalytic activity of MOT11 and MOT14 was measured at room temperature. RhB dye (10 ppm) was dissolved in 100 mL of water. The RhB absorption peak at 554 nm was adjusted as a characteristic peak for monitoring the photocatalytic degradation process. The rate of degradation was found to be zero without photocatalyst, indicating nearly low levels of degradation. Then, at the natural pH, 0.1 mg of photocatalyst was used for each degradation process. Initially, the prepared solution containing dye was continuously stirred for 30 min in the dark to observe homogeneity. Drastic degradation was observed even without solar light irradiation. Afterwards, the degradation was observed after every 30 min.

### 2.7. Photoelectrochemical Measurements

Mott–Schottky (MS) measurements were conducted by using an electrochemical workstation. Moreover, a standard three-compartment cell (consisting of a photo-/working electrode (WE), a Pt wire counter electrode (CE), and an Ag/AgCl reference electrode (RE) with 0.5 M Na_2_SO_4_ electrolyte solution (pH = 4.8) were used. The working electrode was prepared using a doctor blade technique and then annealed at 300 °C for 2 h to remove organic chemicals. Flat-band potentials (V_FB_) were determined by MS measurements performed at a frequency of 1000 Hz with 10 mV amplitude. The V_FB_ of each semiconductor was determined by extrapolating the plot of the inverse square of capacitance (1/C_sc_^2^) arising from the space charge layer in a semiconductor versus applied potential (V).

## 3. Results and Discussion

### 3.1. Structural and Compositional Analysis

**Figure 1:**
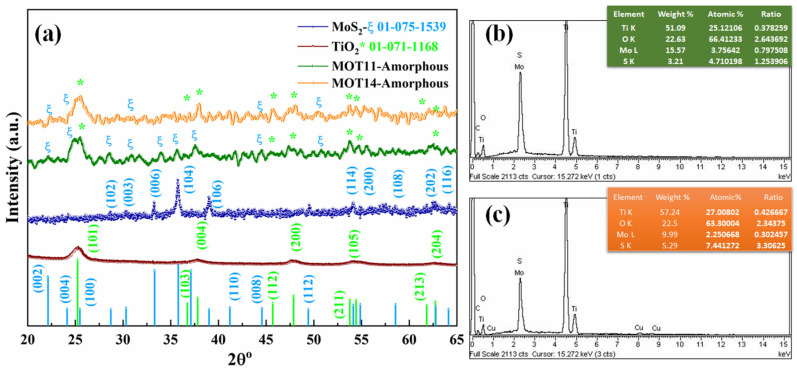
(**a**) XRD pattern of prepared MoS_2_, TiO_2_, MOT11, and MOT14; (**b**,**c**) EDX spectra for MOT11 and MOT14, respectively.

The structural composition of MoS_2_, TiO_2_, and MoS_2_/TiO_2_ (MOT11 and MOT14) heterostructures was examined using the XRD pattern as shown in Figure 1a. The diffraction peaks for MoS_2_ were well matched with the hexagonal phase, ICCSD#01-075-1539 (a = 3.14 Å, b = 3.14 Å and c = 12.53 Å). For TiO_2_ the diffraction peaks were well matched with anatase phase with tetragonal structure, ICCSDs 01-071-1168 (a = 3.79 Å, b = 3.79 Å and c = 9.57 Å). The crystal growth was observed along the (104) plane for MoS_2_ and along the (101) plane for TiO_2_. The lattice constants of MoS_2_ and TiO_2_ were also calculated by using the following Equations (1) and (2) [19,20]:

For

(1) $${MoS}_{2}\frac{1}{d^{2}}=\frac{4}{3}\left(\frac{h^{2}+hk+k^{2}}{a^{2}}\right)+\frac{l^{2}}{c^{2}}$$

and for

(2) $${TiO}_{2}\frac{1}{d^{2}}=\frac{h^{2}+k^{2}}{a^{2}}+\frac{l^{2}}{c^{2}}$$

whereas the above consequences were found to be in good agreement with MoS_2_ (a = 3.12 Å, b = 3.11 Å and c = 11.93 Å) and TiO_2_ (a = 3.89 Å, b = 3.56 Å and c = 9.32 Å), and slight changes were observed due to differences in their morphologies. For heterostructures MOT11 and MOT14, amorphous XRD patterns were observed. The matched planes with MoS_2_ and TiO_2_ are marked in Figure 1a, which indicates the successful development of heterostructures. To further confirm the composition, EDX spectra for MOT11 and MOT14 were provided in Figure 1b,c, respectively. The calculated ratio validated the successful development of MoS_2_ and TiO_2_ heterostructures.

**Figure 2:**
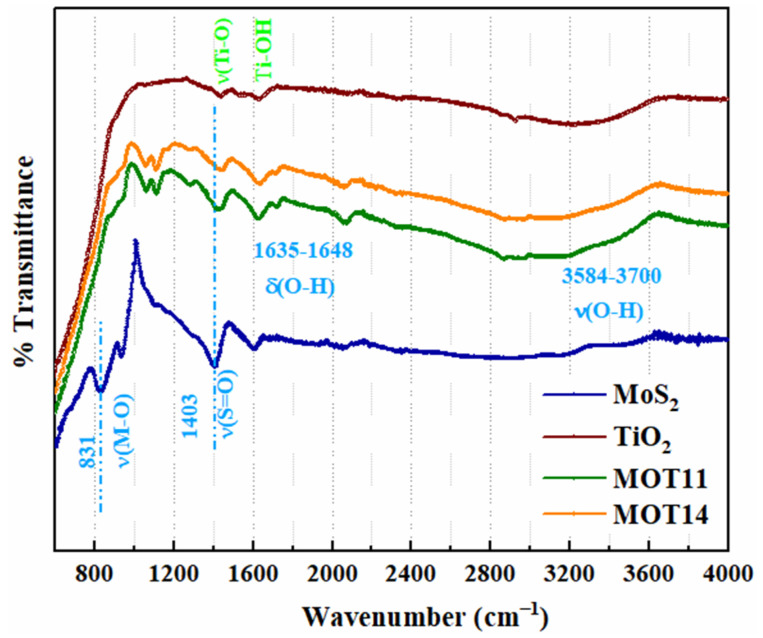
FTIR spectra for MoS_2_, TiO_2_, MOT11, and MOT14.

The FTIR spectra of MoS_2_, TiO_2_, MOT11, and MOT14 were analyzed and shown in Figure 2. The observed peaks were matched with characteristics peaks from the literature, which further confirms the MoS_2_/TiO_2_ heterostructures’ formation. For TiO_2_ absorption, a peak near 1630 cm^−1^ is associated with the bending mode of Ti-OH, and 1383 cm^−1^ is the vibrational frequency of Ti-O [19]. The spectrum of MoS_2_ exhibited absorption peaks at 831 cm^−1^ and 1403 cm^−1^ corresponding to vibrational frequencies of M-O and S=O, respectively [16]. Moreover, the presence of O-H was found at 1635–1648 cm^−1^ and 3584–3700 cm^−1^ indicating the moisture present on the surface [16]. MOT11 and MOT14 represent similar peaks with much broader absorption but at higher transmittance values than MoS_2_ and TiO_2_, such as at 1197 cm^−1^ and 699 cm^−1^; these values are associated with S=O and Mo-O, respectively [13,16].

### 3.2. Morphological Analysis

**Figure 3:**
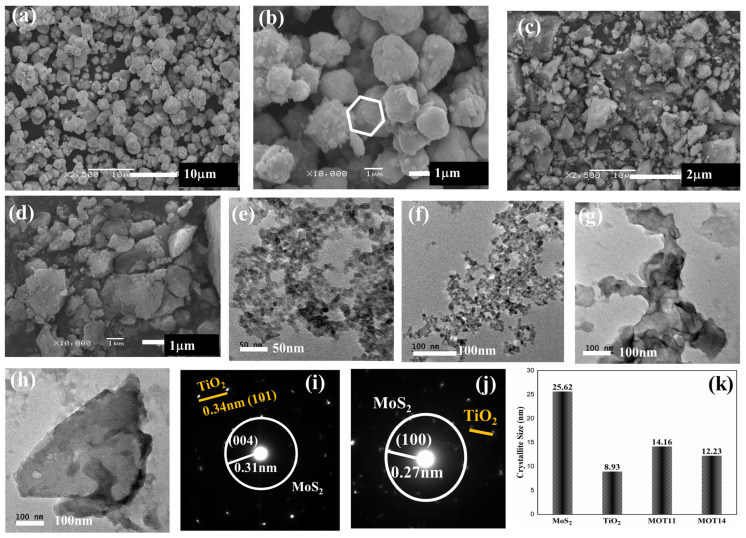
SEM images of MOT11 (**a**,**b**) and MOT14 (**c**,**d**); TEM images of MOT11(**e**,**f**) and MOT14 (**g**,**h**); SAED pattern of MOT11 and MOT14 (**i**,**j**); and (**k**) crystallite sizes of MoS_2_, TiO_2_, MOT11 and MOT14.

The MOT11 and MOT14 heterostructures’ morphology was examined by SEM and TEM, as shown in Figure 3. SEM images of MoS_2_, TiO_2_, MOT12 and MOT13 are provided in Figures S1 and S2 of the Supplementary Information. According to Figure S1, the agglomerated hexagonal structure of MoS_2_ and the layered structure of TiO_2_ can be seen.

For MOT11, the agglomerated hexagonal structure is more visible than the TiO_2_-layered structure in Figure 3a,b. However, in Figure 3c, an organized agglomeration compared to MoS_2_ was observed, possibly due to an equivalent ratio of MoS_2_ and TiO_2_ for MOT11. For MOT14, by increasing the ratio of TiO_2_ by four, the layered structure overwhelmed the agglomerated hexagonal structure, as can be seen in Figure 3d,e. The layered structures can also clearly be seen in the TEM image in Figure 3f. This also led to the structural deformation of the agglomerated hexagonal structures. Moreover, the reduced particle size for MOT11 than MOT14 showed better photocatalytic results, suggesting that the 1:1 ratio of MoS_2_ and TiO_2_ was more favorable than the 1:4 ratio. The morphology results suggest that by further optimization of the synthesis process and ratios, a uniform morphology and crystal structure may be obtained, leading to different photocatalytic results. However, according to our previous study, amorphous materials have shown excellent photocatalytic results [4,23]. SAED patterns for MOT11 and MOT14 are presented in Figure 3i,j, respectively. These patterns further confirmed the formation of heterostructures of MoS_2_ and TiO_2_. Moreover, the crystalline sizes of MoS_2_, TiO_2_, MOT11, and MOT14 were calculated through XRD results, which are provided in Figure 3k. MoS_2_ = 25.62 nm has the largest crystallite size, indicating that its crystalline domains are more extensive compared to the others. TiO_2_ = 8.93 nm has the smallest crystallite size. A smaller crystallite size is typically indicative of a higher surface area, which can be beneficial in applications like photocatalysis. It indicates that TiO_2_ has a more finely divided crystalline structure compared to MoS_2_ and others. This is also evident by the XRD results. MOT11 = 14.16 nm has a crystallite size that falls between MoS_2_ and TiO_2_. It may represent a mixed material or a specific phase of TiO_2_ that is doped or combined with MoS_2_, leading to intermediate properties. The smaller size compared to MoS_2_ may suggest a higher surface area while still maintaining a relatively large crystalline domain. MOT14 = 12.23 nm is slightly smaller than MOT11. Its size suggests it could have a surface area like MOT11, but one that is slightly larger due to the smaller crystallite size. From MoS_2_ to TiO_2_, there is a clear trend of decreasing crystallite size. This suggests that the pure MoS_2_ sample has larger crystalline domains compared to the pure TiO_2_ sample. MOT11 and MOT14 show intermediate crystallite sizes, suggesting a combination of properties from both MoS_2_ and TiO_2_.

### 3.3. UV-Vis Spectroscopy

**Figure 4:**
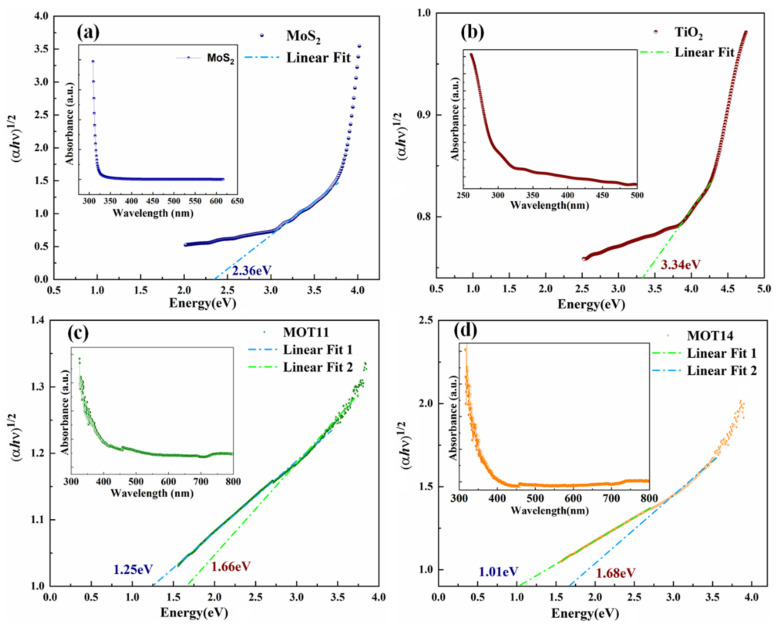
Tauc’s plots for (**a**) MoS_2_, (**b**) TiO_2_, (**c**) MOT11, and (**d**) MOT14, were obtained through the UV-Vis absorption curves shown in the insets.

The UV-Vis absorption spectra of MOS_2_, TiO_2_, MOT11, and MOT14 are measured and the corresponding absorption spectra are provided in the inset of Figure 4 and Figure S4 of the Supplementary Information for clear observation. The bandgap energies were estimated by the linear fit of the Tauc’s plot derived by Equation (3) [23]:

(3) $$\alpha h\nu =A(h\nu -E_{g}{)}^{n}$$

where *n* = 1/2 corresponds to the indirect bandgap, hν  is the photon energy, *A* is a proportional constant, *E_g_* is the bandgap energy, and α  is the absorption coefficient. The estimated bandgaps of MoS_2_ and TiO_2_ are 2.36 eV and 3.34 eV, respectively, as shown in Figure 4a,b. The bandgaps were red-shifted to 1.66–1.25 eV and 1.01–1.68 eV for MOT11 and MOT14, respectively, as can be confirmed from Figure 4c,d. The double bandgaps estimation confirms the successful formation of the heterostructures.

### 3.4. Photocatalytic Degradation

**Figure 5:**
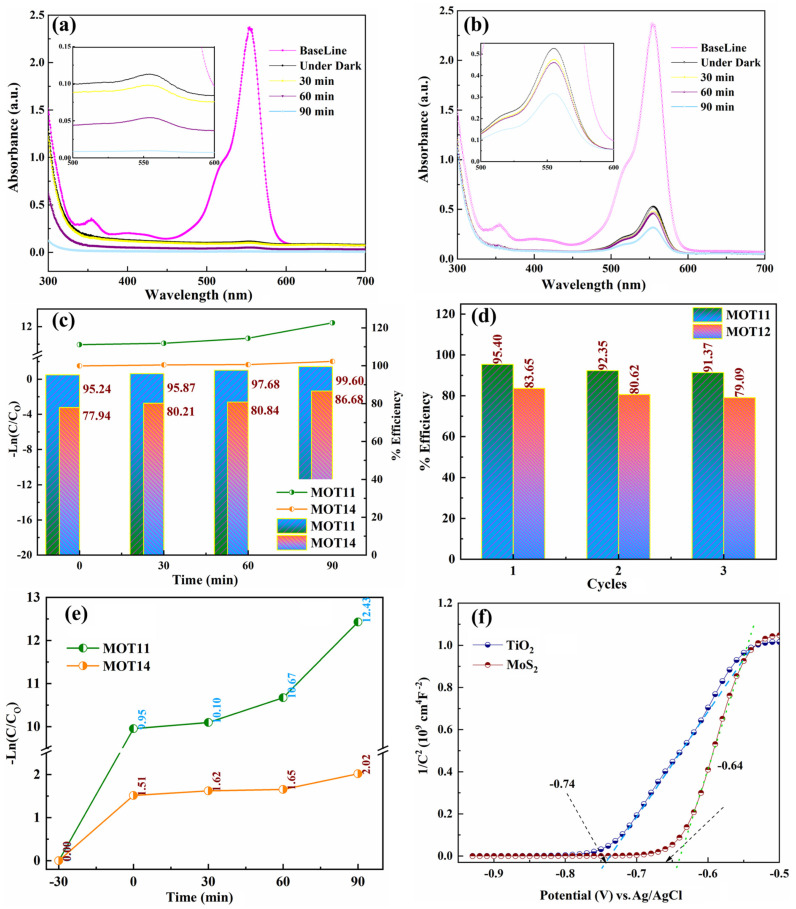
Photocatalytic degradation of RhB under solar light irradiation for (**a**) MOT11 and (**b**) MOT14; (**c**) Pseudo-first-order kinetics (lines with dots) and % Efficiency (bars) with change in time; (**d**) stability graph for MOT11 and MOT14 heterostructures; (**e**) photocatalytic degradation following the first-order kinetics, and (**f**) Mott–Schottky plot for MoS_2_ and TiO_2_ at 1000 Hz vs. Ag/AgCl converted to pH = 7.

The change in absorption of RhB degradation for MOT11 and MOT14 was measured for a fixed time interval of 90 min to understand the degradation kinetics, as shown in Figure 5. The photodegradation efficiency (% Efficiency) of different catalysts was determined using Equation (4) [23,24]:

(4) $$\%Efficiency=(\frac{C_{0}-C}{C_{0}})\times 100$$

where C is the concentration of RhB at time interval t and $C_{0}$ is the concentration after the adsorption equilibrium was established and before irradiation was indicated as the baseline in Figure 5a,b. The characteristic absorption peak of RhB was set at 554 nm. To establish an equilibrium between RhB and photocatalysts (MOT11 and MOT14), the solution was kept in the dark for 30 min. After 30 min of irradiation, RhB almost degraded for MOT11 (Figure 5a and its inset); however, for MOT14 (Figure 5b), a small amount of degradation was observed. After 90 min, complete degradation for MOT11 was observed and 99.60% Efficiency was calculated. However, for MOT14, 86.68% Efficiency was calculated. The detailed variation in the % Efficiency of MOT11 and MOT14 with time was also provided in Figure 5c. The photocatalyst stability was checked for three cycles, as shown in Figure 5d. Accordingly, MOT14 is more stable than MOT11; for MOT11, the stability was dropped to ~7.6% after the third stability test cycle, indicating that it is a stable photocatalyst. MOT14’s stability was dropped to ~8.23% after the third stability test cycle. However, the efficiency of MOT11 was greater than that of MOT14. The degradation followed first-order pseudo kinetics, as shown in Figure 5c–e. Rate constants of 8.29 k/h for MOT11 and 1.34 k/h for MOT14 were calculated, and these were much greater than those of many studied photocatalysts in the literature [15,20,22]. Moreover, the photocatalytic activity of MOT13 and MOT14 is also provided in Figure S4 for comparison.

MoS_2_ and TiO_2_ heterostructures serves as the photocatalyst, enabling these reactions by absorbing light and generating the necessary electron–hole pairs. The reaction kinetics are provided in Equations (5)–(15) demonstrating a typical photocatalytic process. Typically, solar light irradiation is used to generate reactive species, such as hydroxyl radicals (•OH) and superoxide anions (O_2_•¯), leading to the degradation of RhB organic dye. These species (•OH and O_2_•¯) are powerful oxidants that can effectively degrade pollutants into less harmful substances. A detailed explanation with reaction kinetics is presented below:

MoS_2_ + hv → MoS_2_ (excited electrons e^−^) (5)

MoS_2_ (excited electron) + hv → MoS_2_ + heat (6)

TiO_2_ + hv → TiO_2_ (excited electrons) (7)

TiO_2_ (excited electron) + hv → TiO_2_ + heat (8)

According to Equations (5)–(8), when MoS_2_ and TiO_2_ are exposed to light (hv), they absorb energy and generate electron–hole pairs. The excited electrons (e^−^) move from the valence band (VB) to the conduction band (CB), while positive holes (h⁺) are left behind in the VB. Some of the excited e^−^ may lose their energy non-radiatively and return to the ground state, releasing heat instead of contributing to further reactions.

TiO_2_ (excited electron) + MoS_2_ (hole in valence band) → Internal Electric Field (IEF) (9)

An internal electric field was generated after TiO_2_ and MoS_2_ came into contact to form a heterostructure. This electric field was also developed due to the e^−^ present in the CB of TiO_2_ and holes h^+^ of VB of MoS_2_.

MoS_2_ (excited electron) + O_2_ → TiO_2_ + O_2_•¯ (10)

O_2_•¯ + H⁺ → HO_2_• (11)

The excited electron in the CB of MoS_2_ can reduce oxygen (O_2_) to form superoxide anions (O_2_•¯). These reactive oxygen species are crucial for breaking down dyes and other organic pollutants. The superoxide anion (O_2_•¯) can pick up a proton (H⁺) to form a hydroperoxyl radical (HO_2_•), which is another reactive species involved in the degradation process.

TiO_2_ (hole in valence band) + H_2_O → TiO_2_ + OH• + H_2_O (12)

The positive hole (h⁺) in the VB of TiO_2_ interacts with water (H_2_O), producing hydroxyl radicals (•OH) and protons (H⁺). Hydroxyl radicals are highly reactive and play a key role in degrading organic pollutants like dyes. These holes can also react with hydroxide ions (OH⁻) to produce more hydroxyl radicals (•OH), further increasing the system’s oxidative potential.

RhB + excited electron in conduction band → reduction products(13)

RhB + hv → oxidation products(14)

RhB + OH• → degradation products(15)

The RhB dye molecules can accept electrons from the conduction band of MoS_2_, leading to their reduction into less harmful products. Solar light energy (hv) can directly excite RhB molecules, resulting in their oxidation and conversion into other products. The hydroxyl radicals (•OH) generated earlier are highly reactive and attack RhB molecules, leading to their breakdown into smaller, less toxic degradation products.

**Figure 6:**
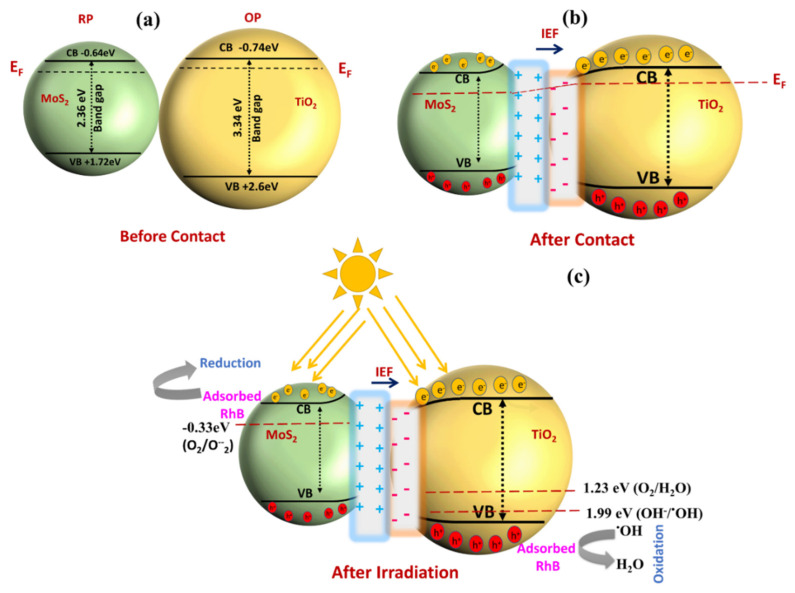
Schematic illustration of RhB photodegradation by MOTs: (**a**) before contact, (**b**) after contact and (**c**) after irradiation.

The typical reaction kinetics are further confirmed by estimating the band potentials of MoS_2_ and TiO_2_ by the Mott–Schottky analysis (MS) provided in Figure 5f. The MS curves were recorded at 1000 Hz vs. Ag/AgCl and converted to pH = 7. According to the measured bandgaps and MS anticipated potentials, the CB and VB energies are −0.74 eV and +1.62 eV for MoS_2_, respectively, and they are −0.64 eV and +2.74 eV for TiO_2_. Based on the band edges’ positions, the charge-transfer process took place in an S-scheme heterojunction between two semiconductors. A schematic illustration of the process is provided in Figure 6.

In an S-scheme heterojunction photocatalyst system, the interaction between an RP and an OP involves a series of processes that enhance charge separation and photocatalytic efficiency. When RP and OP come into contact, their different CB and VB levels, along with their distinct work functions, cause electrons to flow from the RP (which has a smaller work function) to the OP, as shown in Figure 6a. This electron transfer creates an electron depletion zone in the RP and an electron accumulation zone in the OP, leading to a positively charged RP and a negatively charged OP. This charge redistribution establishes an internal electric field directed from RP to OP, driving photogenerated electrons from OP to RP, as shown in Figure 6b. Simultaneously, the contact between RP and OP causes their Fermi levels to align, resulting in band bending at the interface. The Fermi level of the RP shifts downward, while that of the OP shifts upward, creating a gradient that facilitates the recombination of photogenerated electrons from the CB of OP with holes in the VB of RP at the interface, as shown in Figure 6c. This process is akin to water flowing downhill to minimize energy, promoting efficient recombination at the boundary. Additionally, the Coulombic attraction between photogenerated electrons in OP and holes in RP further encourages this recombination, helping to eliminate excess carriers.

To further enhance charge separation, scavengers are often employed in S-scheme systems. Electron scavengers capture photogenerated electrons, particularly from the CB of RP, preventing their recombination with holes. This leaves more holes available in the OP for oxidation reactions, enhancing the system’s overall oxidation capacity. Meanwhile, hole scavengers trap holes from the VB of OP, preventing them from recombining with electrons. This process promotes reduction reactions by enabling more electrons in RP to participate in those reactions [25,26,27].

**Figure 7:**
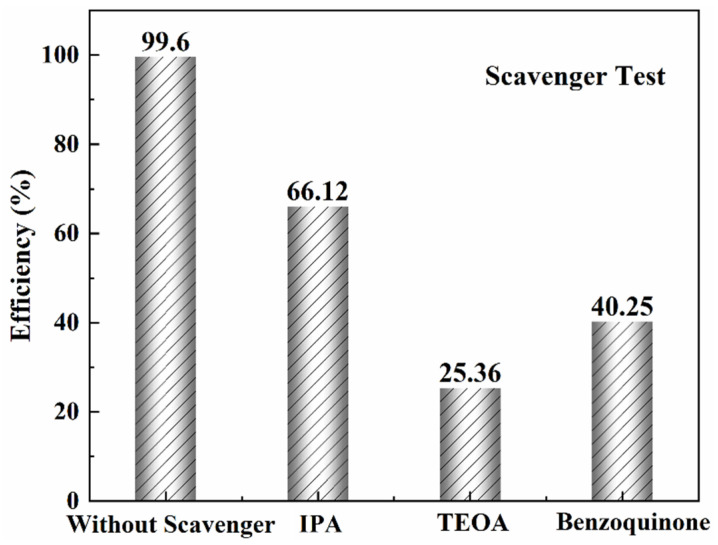
Scavenger test for the photodegradation of RhB to test the active species.

Overall, scavengers play a crucial role in selectively removing electrons or holes, reducing recombination, and supporting the S-scheme’s goal of spatially separating photogenerated charge carriers. This selective capture increases the efficiency of redox reactions, allowing the electrons in RP to drive reduction processes and the holes in OP to facilitate oxidation. Figure 7 represents the role of various scavengers in the “scavenger test” and their impact on the efficiency of a particular reaction or process, likely a photocatalytic reaction. Their absorption spectra are also provided in the Figure S7. Scavenger 99.6% shows the highest efficiency when no scavengers are present, suggesting that the reaction proceeds with minimal hindrance or side reactions. It serves as a control to show the full potential efficiency of the proposed reactions in the reaction kinetics. IPA (Isopropyl Alcohol)—66.12% Efficiency—is often used as a hydroxyl radical (•OH) scavenger. The reduction in efficiency when IPA is introduced indicates that hydroxyl radicals play a significant role in the reaction. Since the efficiency drops to around 66.12%, it suggests that a considerable portion of the reaction relies on the activity of hydroxyl radicals. TEOA (Triethanolamine)—25.36% Efficiency—is typically a scavenger for photogenerated holes (h^+^). The significant reduction in efficiency (down to 25.36%) upon the introduction of TEOA indicates that the reaction is heavily dependent on the participation of holes. This drop suggests that holes are critical in the reaction mechanism; however, they play lesser roles than the •OH radicals, which contribute substantially to the overall reaction efficiency. Benzoquinone—40.25% Efficiency—acts as a superoxide radical (•O_2_⁻) scavenger. The decrease in efficiency to 40.25% when benzoquinone is used suggests that superoxide radicals are also important for the reaction, though less so than hydroxyl radicals and holes. From the scavenger test, it is evident that the efficiency of the reaction is highest when no scavengers are used, suggesting that hydroxyl radicals, holes, and superoxide radicals all contribute to the overall effectiveness of the reactions. The steep drop in efficiency when TEOA is used indicates that photogenerated holes are the most crucial active species. The presence of IPA and benzoquinone also reduces efficiency, but to a lesser extent, implying that hydroxyl radicals and superoxide radicals are also involved in the reaction mechanism.

## 4. Conclusions

MoS_2_/TiO_2_ (MOT) heterostructures were synthesized through a simple physical method using various ratios between the two components. Initially, MoS_2_ and TiO_2_ were prepared separately via hydrothermal synthesis routes. Among the different ratios studied, the optimized compositions, denoted as MOT11 and MOT14, exhibited significantly enhanced light absorption and superior photocatalytic activity. After 90 min of solar light irradiation, the photocatalytic efficiencies were found to be 99.60% for MOT11 and 86.68% for MOT14. The calculated rate constants were 8.29 k/h for MOT11 and 1.34 k/h for MOT14, surpassing those of many previously reported photocatalysts. The remarkable photocatalytic performance of these heterostructures is attributed to the prepared heterostructures of the TiO_2_ nanosheets and MoS_2_ agglomerated hexagonal nanostructures. These facilitate efficient charge separation and transfer, which is further validated through detailed band potential analysis and proposed reaction kinetics within the S-scheme framework. In an S-scheme process, the internal electric field, band bending, and Columbic attraction collectively work to suppress the recombination of photogenerated electron–hole pairs while preserving high-energy electrons in the conduction band (CB) of the reduction photocatalyst (RP) and holes in the valence band (VB) of the oxidation photocatalyst (OP), thus driving the catalytic reactions. Furthermore, scavenger tests were conducted to identify the primary reactive species in the degradation process, revealing that hydroxyl radicals (•OH) and superoxide radicals (O_2_•⁻) are the key contributors. However, the •OH radicals play a dominant role; these are generated by the electrons in the CB and eventually participate in the reduction reactions, further promoting the photocatalytic activity. In conclusion, the prepared S-scheme MOTs demonstrate significant potential for various industrial applications, including pollutant degradation, hydrogen production, and NO_x_ removal, owing to their high photocatalytic efficiency and favorable reaction mechanisms.

## Data Availability

Data are contained within the article or Supplementary Materials.

## Supplementary Materials

The following supporting information can be downloaded at: https://www.mdpi.com/article/10.3390/nano15010028/s1, Figure S1: FESEM images of prepared (a) MoS_2_ and (b) TiO_2_; Figure S2: FESEM images of prepared (a) MOT12 and (b) MOT13; Figure S3: XRD results of prepared MOT12 and MOT13 in comparison with MOT11; Figure S4: RhB photocatalytic degradation by using MOT12 and MOT13 photocatalysts; Figure S5: Absorption spectra of MoS_2_, TiO_2_, MOT11 and MOT14; Figure S6: Photocatalytic degradation following the first-order kinetics; Figure S7: Photocatalytic degradation following the scavenger test after 90 min; Table S1: Specific surface area of the prepared samples.
