# Supplementary material for: Solar Light-Driven Efficient Degradation of Organic Pollutants Mediated by S-Scheme MoS2@TiO2-Layered Structures
Source: Nanomaterials (Basel). 2024 Dec 27;15(1):28. doi: 10.3390/nano15010028 (PMC11722417; doi:10.3390/nano15010028)
Supplement: Supplementary file 1 [file nanomaterials-15-00028-s001.zip › nanomaterials-3297989-supplementary.pdf]

Supplementary Information

Solar-Light-Driven-Efficient Degradation of Organic Pollutants Mediated by S-Scheme MoS_2@TiO_2-Layered Structures

Wajeehah Shahid[1], Faryal Idrees*[2], Ji-Jun Zou[3], Jeong Ryeol Choi*[4], Lun Pan[3,5]

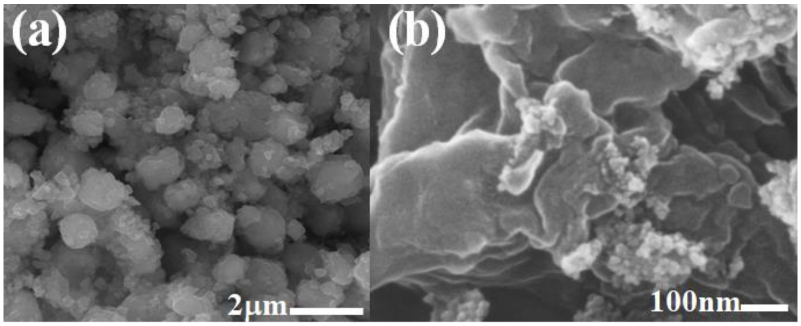

Figure S1. FESEM images of prepared (a) MoS_2 and (b) TiO_2.

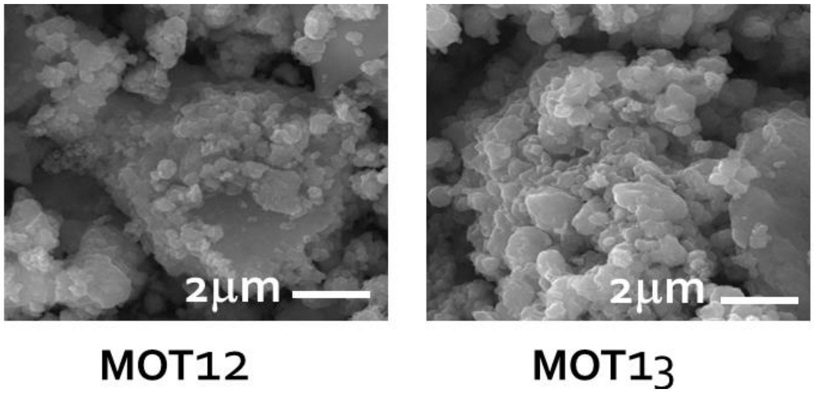

Figure S2. FESEM images of prepared (a) MOT12 and (b) MOT13.

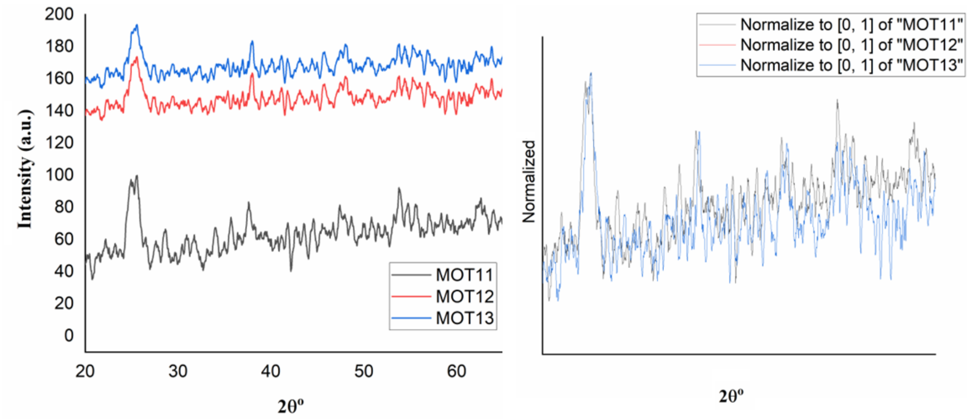

Figure S3. XRD results of prepared MOT12 and MOT13 in comparison with MOT11.

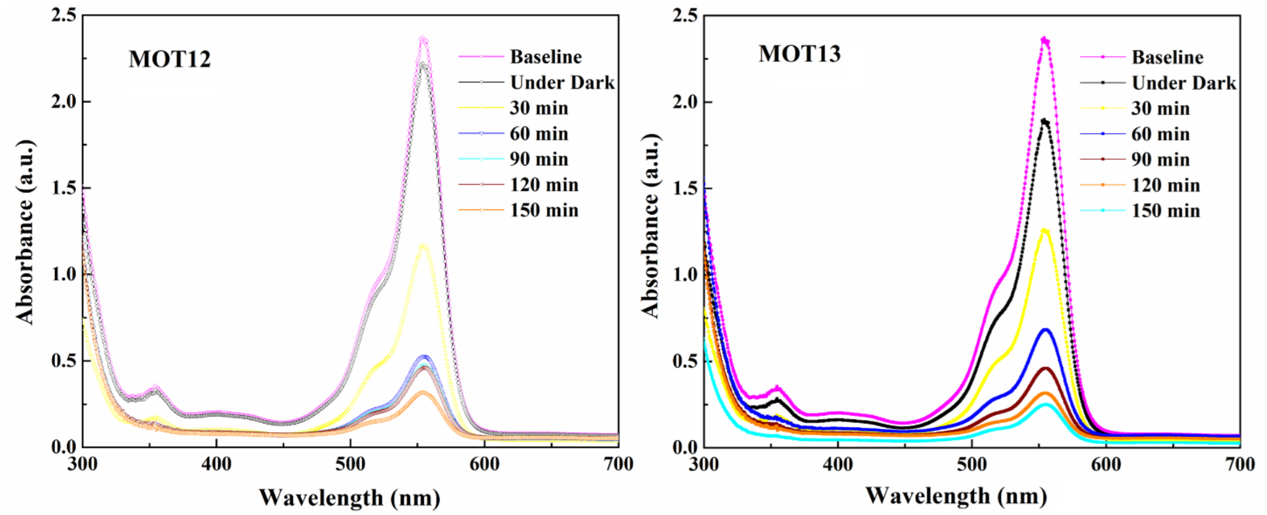

Figure S4. RhB photocatalytic degradation by using MOT12 and MOT13 photocatalysts.

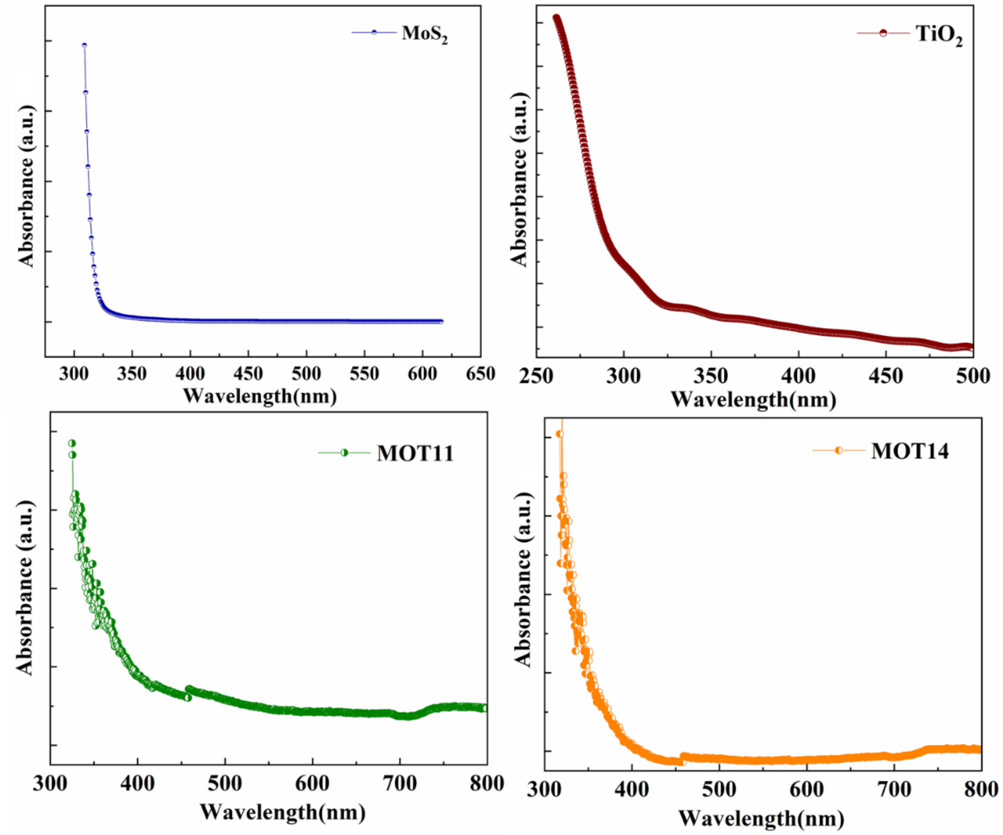

Figure S5. Absorption spectra of MoS_2, TiO_2, MOT11 and MOT14.

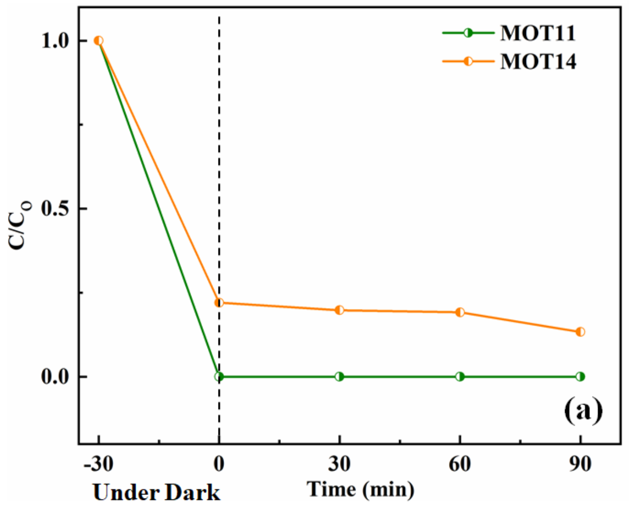

Figure S6. Photocatalytic degradation following the first-order kinetics

Photoelectrochemical conversions calculation for pH=7 are as follows:

$$V_{CB} \approx V_{FB(NHE,pH\,7)} = V_{FB}(\,Ag/AgCl\,pH\,5.9) - 0.059(7-5.9) \qquad \text{Equation S1}$$

$$V_{VB} = V_{CB} + E_g/e, \qquad \text{Equation S2}$$

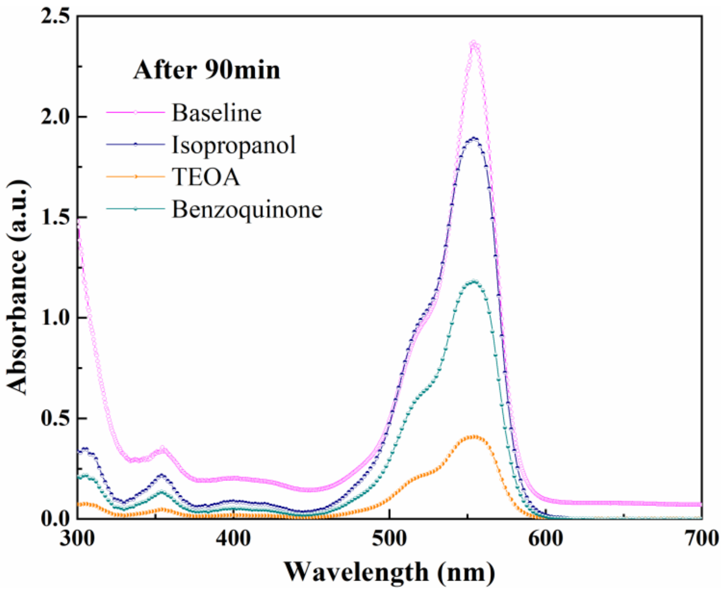

Figure S7. Photocatalytic degradation following the scavenger test after 90 minutes.

Table S1: Specific Surface Area of the prepared Samples

**Samples**	**SSA (m^2/g)**	**Samples**	**SSA (m^2/g)**
TiO_2	169	MoS_2	50
MOT11	150	MOT12	129
MOT13	130	MOT14	160
